# Supplementary material for: Are carbon emissions trading and green financial instruments synergistic? -Comprehensive quantitative research based on content analysis
Source: PLoS One. 2024 Mar 7;19(3):e0298601. doi: 10.1371/journal.pone.0298601 (PMC10919601; doi:10.1371/journal.pone.0298601)
Supplement: S1 Data — (ZIP) [file pone.0298601.s001.zip › Data/地级市Z_F工作报告.zip!/║┌┴·╜¡/╣■╢√▒⌡╩╨/└┤╫╘╣1⁄2╓┌║┼í╛┬φ┐╦╩2╛▌═°í┐_2014.docx]

各位代表：

现在，我代表市人民政府向大会报告工作，请予审议，并请市政协各位委员提出意见。

一、2013年工作简要回顾

过去一年，是哈尔滨发展历程中极不平凡的一年，是有效应对错综复杂经济形势、科学发展基础进一步夯实的一年，也是成功战胜历史罕见特大洪水和暴雪袭击、城市凝聚力和向心力显著增强的一年。我们在省委、省政府和市委的正确领导下，紧紧依靠全市人民，深入贯彻落实党的十八大、十八届三中全会精神和习近平总书记系列重要讲话精神，以及省委、省政府的战略部署，牢牢把握科学发展这一主题和加快转变经济发展方式这条主线，坚持稳中求进的总基调，着力稳增长、调结构、促改革、扩开放、惠民生，锐意进取、扎实工作，开创了全市经济社会发展新局面。

经济综合实力跨上历史新台阶。预计地区生产总值突破5000亿元大关,比上年增长9%左右，社会消费品零售总额增长14%左右。完成公共财政预算总收入632.4亿元，其中，地方公共财政预算收入402.3亿元，分别增长8.8%和13.4%。万元GDP综合能耗下降3.56%以上，经济运行质量和效益进一步提升。

产业项目建设实现新突破。全年开工建设2000万元以上项目1041个，完成投资1300亿元，其中，实施省市重点产业项目277个，完成投资720亿元，分别是上年的1.15倍和1.22倍，开工速度之快、数量之多、投资之大，在哈尔滨产业项目建设史上尚属首次。

经济发展活力不断释放。预计实现固定资产投资5200亿元，增长32%，增速连续五年居全国副省级城市之首。工业投资大幅增长，完成1600亿元，增长33%；民间投资更加活跃，实现3100亿元，增长35%，占总投资比重达59%。国资国企改革持续深化，交通、文旅、住建集团正式挂牌运营，国有资产运营和配置效率明显提升；行政审批制度改革步伐加快，减少行政审批事项30个，优化政府办事流程196个，社会交易成本大幅下降，服务经济发展水平进一步提高。

人民生活持续改善。实现城市居民人均可支配收入25222元、农村居民人均纯收入10800元，分别增长12.1%和14.1%。年初确定的70个惠民行动项目件件落实，完成投资330亿元，超年计划70亿元，全市人民在改革发展中得到了更多实惠。

精神文明与民主法治建设得到加强。文明城市创建和公民道德建设扎实推进，涌现出刘效忠、苏玉双等一大批引领哈尔滨风尚的道德楷模。自觉接受人大依法监督和政协民主监督，人大代表建议、政协提案办复率均达100%。“六五”普法深入实施。创建全国最具安全感省会城市成效显著，刑事案件发案总量保持省会城市最低，公众安全感满意度达93.5%，信访积案化解率达85%，和谐社会建设取得积极进展。

各位代表，过去一年，我们之所以能够顶住经济下行压力，有效克服各种不利因素和风险挑战，保持了经济在合理区间较快增长、各项社会事业协调发展、人民生活水平不断提高，关键是按照党中央、国务院、省委、省政府的战略部署，结合市情，科学施策，扎扎实实推进了以下六个方面工作。

（一）奋力推进产业项目建设，不断培育壮大新的经济增长点。针对经济总量小、发展速度慢的实际，我们坚持把经济转型、加快发展的着力点和突破口放在产业项目建设上，全力推动产业结构优化升级，开创了产业建设提质增效、快速发展的新局面。注重招大引强增后劲。举全市之力开展了产业大招商活动，构建了以各区县（市）、开发区为主体，市直部门齐上阵的工作新格局，全年新引进开工产业项目836个，是上年的4倍，其中100亿元以上6个、50亿元以上8个、10亿元以上60个，完成投资254.4亿元；新引进世界500强企业7家、国内500强企业17家；实际使用外资22.8亿美元、内资1322亿元，分别增长20%和63.2%，总量和增量均居全省第一位。注重调整结构促转型。围绕改造升级传统产业，实施了百威英博120万吨啤酒等亿元以上项目100个，建成投产项目64个，投资12.3亿元的凯斯纽荷兰项目达产后，将成为东北亚最大的农机生产基地。围绕培育壮大战略性新兴产业，实施了中航科工钛合金等亿元以上项目100个，东安艾维欧航空传动系统等67个项目竣工投产，投资19.8亿元的中船重工燃气轮机达产后，可实现我市动力装备制造业质的飞跃。坚持引进与培育并重，持续壮大企业发展实力，全年新增规模以上工业企业150户，经济加快发展的推动力和竞争力不断增强。围绕提升服务业发展层次和水平，新引进开工10亿元以上现代服务业项目152个，投资200亿元的万达文化旅游城建成后，年可接待游客1000万人次，不仅能聚合文化旅游资源、提高产业发展层级，更能催生和促进多种现代服务业繁荣发展。“迷人的哈尔滨之夏”文化旅游活动成效显著，全年实现旅游总收入668亿元，增长20.5%，城市的影响力和美誉度进一步提升。注重集群发展建园区。不断加大园区基础设施投入力度，把园区发展专项资金由3亿元提高到5亿元，重点园区产业集聚力明显增强。高新区新引进科技研发、产业化及配套项目75个，创新驱动发展的功能更加完善；经开区新落户企业1000家，新开工项目115个，装备制造、食品、信息三大主导产业集群发展初具规模；辽宁华塑等46个产业项目进驻宾西开发区，双胞胎饲料等22个项目建成投产；利民医药产业园区新引进项目16个，“北方药谷”专业园建设步伐明显加快。

注重要素保障强支撑。积极拓宽资金来源渠道，市财政投入各类产业发展专项资金25亿元，设立了每年3个亿、三年10个亿的创业投资引导资金，发行企业债券40亿元，引进保险资金30亿元，非公开发行股份募集资金35.4亿元，企业信用融资担保服务中心担保额达122亿元，金融服务实体经济和产业发展能力得到提高。强化人才智力支撑，设立了1亿元且上不封顶的人才引进资金，引进各类产业发展急需人才2410人。推进科技与经济深度融合，集聚科技成果转化项目1709项，实现本地转化837项，高新技术产业增加值占GDP比重提高到13%。通过一年来的创新实践，我们不仅进一步夯实了产业发展根基，更让广大干部职工在产业招商项目建设过程中，转变了思想观念，提高了服务质量，奋勇争先、干事创业的劲头更加高涨！

（二）深入实施富民强县战略，不断激发县域经济发展活力。立足于发挥县域资源优势、支撑城市经济发展，我们持续加大投入力度，有效促进了工业化和城镇化良性互动、城镇化和农业现代化相互协调，县域经济步入了更好更快发展的轨道。农牧业综合生产能力持续提升。粮食生产战胜多种自然灾害再夺丰收，总产量达330亿斤，实现“十连增”。新建扩建规模化牧业养殖场（区）315个，肉蛋奶产量达290万吨。农业种植结构得到进一步优化，“旱改水”、“粮改菜”成效明显，新增水田113万亩、蔬菜3万亩，新建现代农业示范园区（带）115个，粮食高产创建面积发展到1600万亩。县域工业整体实力不断壮大。规模以上工业总产值实现1087亿元，首次突破千亿元，增长38.1%。新上亿元以上产业项目140个，其中，10亿元以上8个，完成投资23.4亿元，是上年的9.4倍。通河、延寿、木兰、方正4个县刚刚结束没有亿元产业项目的历史，去年又新上亿元以上产业项目58个。五常牛家工业园新引进大连环嘉集团再生资源等47个项目，工业总产值实现112亿元，首次突破百亿元，增长25%。新农村和城镇化建设扎实推进。撤并14个乡（镇）、268个村（屯），建设市级新农村示范村50个，完成扶贫开发整村推进68个，开工建设520公里农村公路，交工通车500公里，改造危险桥梁177座，依兰松花江大桥建成通车。经过一年来的不懈努力，县域经济综合实力不断增强，实现生产总值增长13.7%左右，完成地方公共财政预算收入63.5亿元，增长21%，县域经济在加快发展中迸发出更大活力！

（三）全面提升城市品质功能，不断增强城市发展的承载力和吸引力。围绕建设现代化国际化城市目标，我们把规划建设管理城市的着力点，放在了满足城市发展需要、提升形象品质和方便群众生活上。坚持规划引领，努力让百姓生活的城市更有特色。完成城市总体规划和土地利用总体规划修改，形成空港大道等24项规划成果，犹太老会堂等保护建筑再现历史风貌，东西轴线、铁路大院成为城市新的靓丽风景线。完善交通体系，努力让百姓生活的城市更顺畅。新建改造各类道路111条、桥涵27座，三环路江南江北全线贯通，公滨路、三合路等骨干道路和进乡街高架桥、红旗大街跨河桥建成通车，地铁1号线实现载客试运营，已安全载客超过1500万人次，城市现代化立体交通格局基本形成。新建停车场20处，新增停车泊位1.6万个，新建公交首末站30处、公交枢纽站3座，新增大容量环保公交车605台、出租车2000台，百姓出行条件得到明显改善，“公交都市”创建工作走在全国前列。推进源网建设，努力让百姓生活的城市更有保障。新改扩建源网厂站35座，改造主城区供水、排水、电力、供热、燃气、通讯老旧管网1047公里，建设先锋路等易涝片区应急排水工程12处，主城区源网地理信息系统完成信息采集和功能开发，城市源网设施建设日趋完善、管理更加规范、科学、有效。强化综合整治，努力让百姓生活的城市更干净。全面落实区街主体责任，健全长效管控机制，治脏、治乱、治差专项整治行动成效突出，机械化清扫率、生活垃圾无害化处理率分别达60%和85%。坚定实施绿色清冰雪，冬季城市环境明显好于往年。迎战1961年有连续气象记录以来最大暴雪灾害的成功实践证明，只要我们应对科学、措施有力，完全可以在严控融雪剂使用的前提下，确保城市干净整洁、畅通有序，更好地展现冰雪城市的独有魅力和特色风光。严厉打击违建行为，努力让百姓生活的城市更规范。坚持拆违控违同步推进，征收建设拆违先行，凡是认定的违法建筑，一律实行“零容忍”、“零补偿”，累计拆除违章建筑1.7万处、717万平方米，至少为国家减少经济损失180亿元。拆违不仅顺应了民心，更维护了社会公平正义。加强生态文明建设，努力让百姓生活的城市更秀美。全年实施污染减排项目101项，造林13.8万亩，城区新植树木59.2万株、新增绿地250.6公顷。城市污水集中处理率由83%提高到85%，“三沟”基本实现清水入河，松花江断面水质达到功能区划标准。经过全市上下的共同努力，城市的承载功能和形象品位得到较大提升，哈尔滨更干净、更文明、更有序了！

（四）坚定不移扩大对外开放，不断增强经济发展的外向度和牵动力。为了下好对外开放“先手棋”，我们积极抢抓政策机遇，着力在完善开放功能、拓展合作空间上下功夫，沿边开发开放区位优势更加凸显。立足打造对俄开放“桥头堡”和“枢纽站”，启动编制了内陆港、综合保税区和临空经济区发展规划，对外开放的产业布局和功能定位更加清晰。综保区土地调整方案已获国土部批复，“两头在外”产业集聚效应开始初显，俄罗斯西格玛集团小型飞机、哈飞复合材料转包生产等项目正在争取入驻园区。哈东现代物流产业带启动建设，华南城等重大商贸物流项目建设进展顺利。哈西群力及联络空间实现资源整合，构建了区域开发建设管理新机制，为辟建临空经济区创造了有利条件。立足建设沿边开发开放大通道，开通了直飞美国阿拉斯加包机、对俄航空客货混载业务和国内首条对俄航空货运专线，成立了中俄跨境金融服务中心，设立了哈尔滨国际邮政互换局，建立了中国北方电商物流仓储发寄中心，可通过俄方设在新西伯利亚和叶卡捷琳堡的两个大型保税库辐射俄罗斯全境。立足实施“引进来、走出去”战略，利用市场换项目，成功引进中丹智能供热节能循环产业园，为更新改造老旧供热管网、保障城市供热安全、助推相关产业发展提供了有力支撑；成功举办哈洽会、农博会、寒博会等国际展会，城市知名度和对外影响力进一步提升；与白俄罗斯签订了“一城对一国”战略合作协议，在中白工业园内规划建设哈尔滨园，为在哈企业更好地开拓独联体及欧洲市场搭建了平台，构建了全方位扩大对外开放的新格局。

（五）千方百计为群众办实事办好事，不断使改革发展成果更多更公平惠及全市人民。我们始终把更好地满足人民生产生活需求作为政府工作的出发点和落脚点，着力解决事关人民群众切身利益的住房、就业、社保、教育、医疗等大事实事，进一步增进了全市人民福祉。围绕改善居民住房条件，开工建设各类保障房2.8万套、竣工4.2万套；为1.5万户困难群众提供廉租住房保障；改造农村泥草房7.2万户、498万平方米；实施既有建筑节能改造310万平方米。围绕促进创业就业，成功举办哈尔滨创业（就业）博览会，城镇新增就业13.8万人，登记失业率控制在4.3%以内，转移农村劳动力143万人。围绕提高社会保障能力，持续扩大各类保险覆盖面，8.3万被征地农民养老保险遗留问题得到破解，企业退休人员基本养老金月均增加228元；城乡最低生活保障标准提高到每月460元和每年2508元；新增养老床位2386张，建成社区日间照料服务中心和农村居家养老服务站699个。城镇居民医保政策实现全覆盖，基层医疗卫生机构实施基本药物制度，新农合统筹区域内政策性报销比例由74%提高到81.4%，22种重大疾病省（市）报销比例达70%。人口自然增长率为2.69％。。围绕强化公共服务，新改扩建公办幼儿园80所，配建新区义务教育学校10所，创建义务教育标准化学校和素质教育特色示范学校64所。哈尔滨大剧院、音乐厅建设进展顺利，城区健身设施和城乡公共文化服务网络实现全覆盖。新建千米以上城市社区20个、农村示范社区19个，为街道办事处、乡镇及社区补助财政资金1.2亿元，社会服务功能和基层政权建设得到进一步加强。通过连续实施惠民工程，广大群众在改善生活条件、提高生活质量的喜悦中，对我们这座城市更有信心了、幸福感更强了！

（六）大力推进作风建设，不断提高政府行政效能和服务水平。按照“照镜子、正衣冠、洗洗澡、治治病”的总要求，扎实推进党的群众路线教育实践活动，形式主义、官僚主义、享乐主义、奢靡之风和文山会海、公款吃喝、不作为、乱作为等不良风气得到有效遏制。集中解决物业管理、冬季供暖、产权证办理、棚改回迁、内涝居民安置等群众反映突出的现实问题，制定了物业收费、管理、准入等10项制度和标准，创建市级以上物业管理示范小区233个，维修老旧住宅756万平方米，清除不合格物业企业77家，物业管理年活动取得初步进展和成效；拆并分散燃煤小锅炉316台，新增集中供热能力2500万平方米，居民供热市长热线投诉率下降49.3%；妥善解决了6万户居民房屋产权证办理遗留难题；长期在外临时居住的9439户棚改居民喜迁新居；道外北岗、向江社区创造了内涝避险搬迁户数最多、安置最好、速度最快的历史。严格落实中央和省、市委改进作风规定，严厉查处破坏经济发展环境相关案件145起，问责公职人员315人，经济发展环境得到进一步改善，群众对政府行政效能的满意度不断提升。

各位代表，过去一年取得的成绩，是省委、省政府和市委正确领导的结果，是全市各级领导班子团结奋进、扎实工作的结果，是人大代表、政协委员及中省直单位和社会各界鼎力支持、全力配合的结果，是全市人民拼搏进取、艰苦奋斗的结果。在此，我代表市人民政府，向所有关心支持哈尔滨事业发展的同志们、朋友们，向广大驻哈解放军指战员、武警官兵，致以崇高的敬意和衷心的感谢！

在肯定成绩的同时，我们也清醒地看到，全市经济社会发展还存在一些不容忽视的问题，主要是抵御市场风险能力还不强，受外部环境趋紧、市场需求不振以及我市产业基础薄弱、发展方式粗放、长期积累的体制性、结构性矛盾等多种因素叠加影响，地区生产总值、规上工业增加值未能完成全年预期；经济市场化程度低，资本、土地、技术、人才等市场体系发育不健全，企业利用资本市场直接融资的意识和能力还不强；国有及国有控股企业带动作用不突出，非公经济对经济增长和财政税收的贡献率，与发达地区相比差距仍然很大；城乡居民收入和社会保障能力在副省级城市中仍处于较低水平，物业管理等百姓关注的热点难点问题还没有得到根本解决，改善民生的任务十分艰巨；政府职能转变不到位，职责交叉、推诿扯皮、效率低下等问题不同程度存在；部分公务员作风飘浮、能力不足、抓落实不到位，腐败行为和损害群众利益问题还时有发生。对此，我们要高度重视，切实采取有效措施，认真加以解决。

二、2014年主要任务

2014年是全面深化改革的起步之年，也是完成“十二五”规划任务目标的关键一年，更是哈尔滨经济社会发展站在新起点，推动产业发展、城市建设、民生改善向更高层次迈进的重要一年。

站在新起点，哈尔滨加快发展的机遇更加有利。党的十八届三中全会做出的全面深化改革决定，更加注重经济、政治、文化、社会、生态文明制度创新和政策集成创新，有利于我们进一步凝聚共识、汇集力量，在破除体制机制障碍、增强经济发展内生动力上实现更大突破。国家实施的老工业基地振兴新十年规划，更加注重经济转型和结构升级，有利于我们在提升产业发展质量和效益上迈出更大步伐；实施的“两大平原”农业综合配套改革试验，更加注重调整农业生产关系，有利于我们在统筹城乡一体化发展上取得更大成效；实施的沿边开发开放规划，进一步明确了哈尔滨区域中心城市地位，有利于我们在扩大对俄开放上争得更大主动。省委、省政府高度重视省会城市龙头作用，有利于我们在率先发展、跨越赶超上获得更大支持。只要我们能够抓住机遇，顺势而为，乘势而上，哈尔滨就一定会在抢占先机中拓展更大的发展空间！

站在新起点，哈尔滨加快发展的方向更加明确。中央稳中求进、改革创新的总基调、总要求，提升经济发展质量和效益的明确导向，让我们的思想更加统一、目标更加明确、行动更加自觉。省委、省政府“建设十大重点产业、实施十项重点民生工程”的战略部署，坚持投资拉动和创新驱动的发展措施，使我们的信心更加坚定。市委、市政府持续抓产业、促发展、惠民生打下的坚实基础，实施新战略、实现新跨越的清晰思路和奋斗目标，稳增长调结构促改革的明确任务和务实举措，为我们实现更好更快发展增添了动力、指明了方向。只要我们咬定青山不放松，持之以恒抓推进，坚定不移促落实，哈尔滨就一定会在改革开放、科学发展的道路上阔步前行！

站在新起点，哈尔滨加快发展的愿望更加强烈。越来越多的哈尔滨人，在改革开放的生动实践中，看到了未来，充满了希望；越来越多的哈尔滨人，在产业发展、城市建设、民生改善的成果变化中，坚定了信心，激发了斗志；越来越多的哈尔滨人，在百舸争流、竞相发展的大潮中，找到了差距，感受到了危机，改革意识、进取精神体现得比以往任何时候都更加明显，不甘落后、奋起直追的意愿比以往任何时候都更加迫切，支持改革、投身发展、共创美好生活的期盼比以往任何时候都更加强烈。只要全市上下同心同德、步调一致、奋发图强，哈尔滨就一定会谱写出更加美好的新篇章！

按照市委十三届五次全会要求，政府工作的指导思想是：认真贯彻落实党的十八大、十八届三中全会和习近平总书记系列重要讲话精神，按照中央和省委、省政府的战略部署，牢牢把握主题主线和稳中求进总基调，把改革创新贯穿到经济社会发展的各个领域各个环节，以落实“五大规划”为引领，着力在调结构转方式、加快城乡发展一体化、提高保障和改善民生水平、完善城市承载功能、提升城市文化软实力、建设生态文明城市上取得新突破，在全面深化改革中奋力开创哈尔滨科学发展新局面。

经济社会发展的主要目标是：重点领域和关键环节改革取得突破性进展，经济持续健康发展，城乡居民生活水平有新的提高，生态文明城市建设取得明显成效，力争跨入全国文明城市行列。主要经济指标是：地区生产总值增长8.5%左右；固定资产投资增长26%左右；规模以上工业增加值增长10%左右；社会消费品零售总额增长13.5%左右；地方公共财政预算收入增长9.5%左右；进出口总额增长8%；城市居民人均可支配收入增长10%以上，农村居民人均纯收入增长12%；城镇居民登记失业率控制在4.3%以内；万元GDP综合能耗下降3.48%以上;人口自然增长率控制在4.8％。以内。为实现上述思路目标，着重做好以下工作：

（一）凝聚更大力量推动产业发展和经济转型。坚持把打造经济升级版的立足点放在构建现代产业体系上，扎实开展项目建设质量效益年活动，通过做大增量、盘活存量、提升质量，再造经济发展新优势。大上项目，壮大优势产业。着力打造绿色食品产业，突出抓好34个亿元以上省重点食品产业项目，叫响“寒地黑土”品牌，确保食品工业总产值两年内实现2000亿元。着力打造高端装备制造业，突出抓好重大技改项目100个、战略性新兴产业项目100个，新增规模以上工业企业100户，力争把装备制造业打造成第二个千亿元产业。着力打造云计算产业，突出抓好中移动和中联通云基地等大项目，更多引进基础设施服务、平台服务和软件服务项目，明显提升中国云谷集聚功能，力争把云计算、物联网、电子商务等信息产业打造成第三个千亿元产业。着力打造现代服务业，加快发展金融、现代物流、信息服务、科技研发和技术创新、商务会展等五大生产性服务业。发挥创业投资引导资金作用，更多引进和培育风险投资、创业投资等私募基金，支持哈尔滨银行等符合条件的企业上市融资，鼓励具备条件的民间资本依法发起设立中小型银行。加快发展现代物流业，重点推进哈东现代物流产业带和华南城、集装箱中心站等大项目建设。利用创建国家电子商务示范城市政策机遇，积极申请国家对俄电子商务试点，培育和引进电子商务骨干企业，着力打造第三方电子商务综合服务平台。高质量办好中俄博览会、农博会、寒博会等品牌展会，推动会展经济向国际化、规模化方向发展。突出冰雪、欧陆、金源等文化特色，加快万达文化旅游城、环球海洋世界、金龙山国际旅游度假区等项目建设，全力办好冰雪节、“迷人的哈尔滨之夏”文化旅游活动，全年实现旅游总收入增长20%以上。做强园区，促进产业集聚。把园区发展专项资金提高到6亿元，重点支持产业园区基础设施建设。经开区重点发展先进装备制造、绿色食品和信息产业，开工建设统一食品产业园等项目100个，确保凯斯纽荷兰等60个项目竣工投产。高新区重点依托科技创新城，加快推进中凌信息哈尔滨基地等20个项目建设，确保国际农业科技创新中心等52个项目投产运营。利民开发区立足建设国家级生物医药产业基地，重点抓好国药物流等35个项目，确保哈药总厂等16个项目建成投产。宾西开发区围绕打造哈东地区经济发展强势板块，突出抓好奥瑞德光电三期等30个项目，确保垦丰种业等15个项目建成投产。力争双城开发区晋升为国家级开发区、五常牛家工业园产值突破150亿元。创新驱动，引领产业发展。突出企业创新主体地位，健全科技创新市场导向机制，加快推进科技招商、科技人员创业和产学研协同创新，促进更多的科技创新成果资本化、产业化，着力培育和储备一批科技创新型上市资源。实施“科技创业引导专项计划”，切实增强科技创新对产业发展的支撑力。扩大招商引资，做大产业规模。进一步完善产业大招商工作机制，着力开展定向招商、以商招商、专业招商、板块招商，更多引进投资强度大、核心竞争力强、产业带动作用突出的大项目好项目。全年新引进投资超100亿元产业项目5个以上、50亿元11个以上、10亿元72个以上，实际使用外资、内资分别增长15%和20%。产业建设事关我市长远发展和百姓福祉，我们要始终牢牢抓住不放，以“功成不必在我”的胸怀和更加扎实的产业基础，助推哈尔滨实现科学发展新跨越！

（二）持续推动县域经济加快发展。牢牢把握健全城乡一体化体制机制改革方向，着力在构建以工促农、以城带乡、工农互惠、城乡一体的新型关系上迈出更大步伐。大力推动农村综合改革。创新农业生产经营体制，抓好方正、阿城农村土地承包经营权确权登记颁证国家试点，加快建立市、县、乡、村四级农村土地流转服务平台和土地纠纷仲裁机构，鼓励承包经营权在公开市场上向农民合作社、家庭农场、专业大户、农业企业流转，鼓励农民以土地承包经营权入股发展农业产业化经营，支持阿城等区县与农垦开展场区（县）共建。稳妥推进农民住房财产权抵押、担保、转让，不断增加农民财产性收入。建立农村产权流转交易市场，推动农村产权流转交易公开、公正、规范运行。创新农业服务机制，支持农业院所、农民合作组织开展农技推广，抓好农业示范基地标准化生产、食品安全监管追溯体系和畜牧大县无规定疫病区建设，组建哈尔滨村镇银行，搭建农牧业融资担保平台，农业保险覆盖面扩大到1200万亩。深入实施产业强县工程。坚定不移走以资源换产业、以市场换项目之路，加快发展食品制造等八大产业、乳制品等三大养殖加工板块和十大特色产业基地，突出抓好五常金福粮油、尚志汇源果蔬、通河北药、依兰30万吨稻谷加工等项目建设，做大做强双城雀巢、宾西牛业等50户龙头企业，确保县域工业占全市工业比重提高5个百分点。继续推进“巴木通方依”区域一体化发展，加快形成要事共商、资源共享、产业共建发展新格局。扎实推进农业现代化。坚持以保障国家粮食安全为使命，突出抓好粮食产能稳定增长工程，新建五常水稻、双城玉米等现代农业示范园区（带）60个，粮食总产稳定在330亿斤以上。抓好“菜篮子”工程，全市蔬菜面积扩大到175万亩，畜产品总量提高到310万吨，确保市场供应充足、物价稳定。大兴农田水利，修复重点水毁工程85处，新建续建沿江泵站19处。坚持用现代物质条件装备农业，新增农机具4500台（套），农业综合机械化率提高到88%以上。积极稳妥扎实推进城镇化。坚持以人的城镇化为核心，以创新城镇化健康发展体制机制为牵动，重点编制新型城镇化建设规划，积极探索建立多元可持续资金保障机制，有序推进农业转移人口市民化，切实提高城镇化建设质量和水平。坚持城镇化与新农村建设同步发展，突出抓好南岗、香坊、方正、通河、木兰整区县推进，宾县居仁等10个整镇推进，68个扶贫开发整村推进。完成农村饮水安全工程398处，解决23.8万人安全饮水问题。开工建设木兰松花江大桥，启动莫延公路延寿至宾县段前期工作，推进哈双北线至吉黑高速段公路建设，新建农村公路300公里，通车200公里，改造危桥130座。县域是我市未来发展的广阔空间。我们要借助“两大平原”现代农业综合配套改革试验有利契机，以更加务实的举措和创新实践，让县域经济在竞相发展中，成为推动全市经济社会发展的一支重要力量！

（三）努力抓好城市规划建设和管理。牢牢把握现代化国际化城市发展方向，全面提升城市形象品质，努力让城市环境更具吸引力和竞争力。强化规划引领。按照国家和省打造“哈长”城市群和哈尔滨大都市圈的战略部署，结合新一轮城市总体规划修改调整，着力优化城市空间结构和产业布局，不断提高控详规划的覆盖面和控制性。充分发挥规划龙头作用，加大规划的执行力度，切实维护规划的严肃性和权威性。结合实施“中兴”战略和老城区更新改造，科学规划道外中华巴洛克等历史街区，恢复圣母守护教堂等经典建筑，推动文化、建筑与产业融合发展，进一步传承历史文脉，彰显城市特色。完善城市功能。坚持需求导向、效益优先原则，延伸打通一批主干街路，实施三环、四环互通工程和出城口道路建设，配套推进城区干道支线“微循环”畅通工程，新增道路100公里。建设地铁1号线三期、2号线一期和3号线，启动规划哈阿轻轨，推进哈西地铁联络线工程。积极配合铁路、航空部门，抓好滨北公铁两用桥、哈站改造、哈齐客专、哈佳哈牡快速铁路、太平机场扩建等项目建设，加快构建辐射全省的现代化交通体系。规模实施主城区老旧管网改造，完善智慧管网信息系统，新建改造各类源网厂站33座、管网1000公里。编制全市供热分区规划，逐步实现供热联网运行，启动松花江供水工程，确保城市供热供水安全。深入开展“公交都市”创建活动，新建停车场21处，新增停车泊位1.2万个，新增、更新公交车辆600台，启动建设1座大型公交枢纽站，建成10处公交首末站，开展出租车电召服务，努力让城市交通秩序更规范、百姓出行更方便。加强城市管理。突出区街责任主体作用，积极落实长效机制，持续提高机械化作业能力，城区机械化清扫和清冰雪率均提高到70%。大力整治城乡结合部、市场摊区等脏乱差环境，进一步建立科学规范的垃圾管理体系，严厉查处乱停乱放等交通违法违规行为，确保城市面貌实现更大改观。始终保持拆违控违高压态势，最大限度维护社会公平正义和城市建设秩序。继续开展“物业管理年”活动，深入整治老旧社区环境，维修老旧住宅700万平方米，创建市级以上物业管理示范小区150个，努力让百姓居住的环境更优美、更舒心。促进新区融合发展。统筹新老城区规划建设，全力打通黄家崴子路、江北东环路等一批主干道路，同步推进基础设施有序衔接、联网运行。继续完善城市新区生产生活服务功能，推动教育、医疗等公共服务向新区延伸，创造条件让新区汇聚更多的人流物流资金流。打造干净文明有序城市，是政府肩负的重大职责。我们要以更加科学的态度和卓有成效的管理，让哈尔滨这座现代城市焕发出更加清新、靓丽、迷人的风采！

（四）更加有效地加强生态文明建设。实行最严格的源头保护制度、损害赔偿制度、责任追究制度，着力打造天蓝、地绿、水清美好家园。全力整治大气污染。突出抓好大气污染防治规划编制，着力构建政府统领、企业施治、市场驱动、公众参与的大气污染防治新机制。加强燃煤质量检测，提高集中供热普及率，拆并分散燃煤小锅炉240台，新增集中供热能力1500万平方米；投入3亿元资金，重点治理机动车尾气，确保年内淘汰黄标车4.5万辆；强化大气污染物排放管控，专项整治烟尘、秸秆焚烧，确保空气污染源头治理取得明显成效。着力改善水环境。持续加大松花江流域水污染防治和阿什河综合治理力度，重点启动群力二期污水处理工程，确保朝阳镇等3个污水处理厂通水调试。全面推行“一河一策”治污模式，严格落实水质严重超标流域限批政策，确保水生态环境得到较大改善。扎实推进节能减排。坚持总量减排与质量改善相同步，确保二氧化硫、氮氧化物、化学需氧量和氨氮排放总量比2010年分别减少2%、2.9%、6.4%和9.6%。实施节能与排放双重监控，加快淘汰落后产能。扎实推进哈尔滨国家环保科技产业园建设，使其更好发挥对俄环保技术与产业合作示范基地作用。强化生态环境保护。改革生态环保管理体制，加快建立体现生态文明要求的目标体系、考核办法和奖惩机制，真正把生态保护纳入法制化、制度化、规范化轨道。严守生态保护红线，完成《哈尔滨市主体功能区规划》和6个国家重点生态功能区保护规划，确保生态资源得到有效保护、合理利用。开展环境污染第三方治理，通过向社会购买公共服务，实现生态环境更好保护。加大水土流失治理力度，新增治理面积36万亩，造林9.3万亩，城区新植树木50万株、新增绿地102公顷。建设生态文明、打造美丽哈尔滨，功在当代、利在千秋。我们要以更加严格的保护制度和更加自觉的文明行为，努力让哈尔滨青山绿水永续利用、代代相传！

（五）全面深化改革扩大开放。大力推动各类要素有序自由流动、资源高效配置、市场深度融合，努力为实现哈尔滨科学发展新跨越提供强大动力和体制保障。扎实推进重点领域和关键环节改革。率先做好政府改革这篇文章，全面正确履行政府职能，深化行政审批制度改革，建立行政审批项目评估清理机制，实行审批项目清单制管理，加快推进行政审批实体大厅、网上虚拟大厅和服务云平台建设，全部取消市本级自设行政审批事项，切实优化审批流程、提高审批效率。进一步下放事权，该由区县（市）管理的一律下放到区县（市），该由市场调节的全部放开，真正把该放的权力放到底，把该管的事情管到位。全面优化政府组织结构，积极推进食品药品监督管理体制改革，加强基层监督执法和技术力量；加快整合卫生行政部门、人口和计划生育部门的机构及职责，加强基层医疗卫生和计划生育服务资源配置；积极推广市物价局改革经验，科学调整内设机构，着力解决部门职责交叉、效率低下的问题，确保权责一致、管理高效，确保机构编制只减不增。抓好事业单位分类改革，推动公办事业单位与主管部门理顺关系和去行政化，加快中介机构与行政主管部门脱钩。继续深化国资国企改革，进一步完善国有资本管理体制，加快构建国资委、国有资本投资运营公司、国有企业三级国有资本资产管理经营模式。科学确定国有企业集团功能定位，支持哈投、创投、文旅、哈药等一般竞争性国有企业集团，通过资产整合重组、发展混合所有制经济等方式做大做强，促其在城市建设、产业发展等方面发挥更大作用；推动物业供热、市政、地铁、交通、住房等承担公共服务职能的国有企业集团，加快政企分开、政资分开，实行特许经营、政府监管，通过建立健全经济效益和社会效益相结合的考核办法，促其更好地服务社会、加快发展。着力控制和化解政府性债务风险，把政府承担偿还的债务分门别类纳入财政预算统筹管理，严格举债程序，切实提高土地运作能力和水平，充分发挥土地在资金平衡、防范政府性债务风险上的重要作用。加快构建开放型经济新优势。在继续巩固传统市场、开拓新兴市场的同时，全面实施沿边开发开放战略规划，努力把哈尔滨打造成对俄合作中心城市。突出抓好综合保税区、临空经济区申建、内陆港与铁路集装箱中心站整合，积极探索建立中俄（哈尔滨）自由贸易区。以陆海联运为基础，扩展对俄航空客货混载业务，开通北美航空货运航线，启动对俄电商物流运营，着力打造面向俄罗斯、辐射东北亚、承接北美地区的国际货运通道。加快推进中俄木材交易中心、诺林化工物流中心、黄金珠宝基地、科技合作基地建设，促进对俄经贸科技合作实现战略升级。围绕打造对俄旅游先行示范区，积极争取俄罗斯在哈设立领事机构，力争太平国际机场享有外国游客72小时过境免签政策，为全方位扩大交流奠定坚实基础。加快中白哈尔滨工业园规划建设，为更多的企业“走出去”搭建平台、创造条件。最大限度激发非公有制经济活力和创造力。制定出台扶持中小企业发展政策，鼓励非公企业参与市属国有企业、集体企业改制重组，支持民间投资进入基础设施、金融服务、公共事业等领域，支持中小微企业通过发行私募债、股权转让等方式进行债券、股权融资。改革永无止境。只要我们始终沿着社会主义市场经济改革方向奋勇前行，就一定能够开拓更加广阔的发展前景！

（六）更好地保障和改善民生。着力解决人民群众最关心最直接最现实的利益问题，确保社会既充满活力又和谐有序。全年重点实施10个方面、66个惠民行动项目，完成投资303亿元。

优先发展教育事业。教育事关孩子成长，事关千家万户，必须持续加大投入，促进各级各类教育协调发展，全力办好人民满意教育。进一步加强教育基础设施建设，全年创建素质教育特色学校、义务教育标准化学校57所，完成14所学校危房改造和75所学校供热改造任务，切实改善办学条件。统筹义务教育资源均衡配置，开展区域内校长教师交流轮岗试点，将省重点高中统招生配额比例提高到60%，外来务工和外籍人员随迁子女全部就读公办学校，最大限度促进教育公平。坚持政府统筹、行业主导，加快编制出台职业教育发展规划和支持政策，进一步整合职业教育资源、创新发展机制，加快构建具有哈尔滨特色的现代职业教育体系。

抓好创业带动就业。就业乃民生之本。我们要实施更加积极的促进和稳定就业政策，加快建立政府激励创业、社会支持创业、劳动者勇于创业的新机制。深入推进高层次人才、自主择业转业军官、高校毕业生、失业人员和农民五大群体创业工程，完善就业公共服务保障体系，确保全年新增城镇就业11.7万人，转移农村劳动力145万人。统筹推进就业技能培训、岗位技能提升培训和创业培训，全年培训高校毕业生9000人、失业人员4万人、农民工5万人。全面加大公益性岗位开发和购买力度，努力为高校毕业生等青年群体创造更多的就业机会，使他们在推动经济社会发展中找到自己人生的舞台、发挥更大的作用。

改善城乡居民住房条件。住有所居是人民群众的最大期盼。我们要继续加大建设和供给力度，开工建设保障房2.25万套、竣工1.6万套，改造农村泥草房5万户、320万平方米；启动建设棚改项目47个、145万平方米，惠及棚改居民1.9万户，努力让城乡困难群众圆好“安居梦”、增加更多的财产性收入。

构建更加公平可持续的社会保障制度。社会保障是民生之基、安全之网。我们要以保基本、兜底线、促公平、可持续为准则，持续扩大社会养老保险覆盖面，放宽灵活就业人员参保条件，允许农村、外省户籍、进城务工人员参加基本养老保险，享受转移接续政策。稳步提高基本养老金保障水平，妥善解决被征地农民养老保险遗留问题。适时启动城乡低保标准动态调整机制，切实保障困难群众基本生活。扎实做好受灾群众救助工作，突出抓好6800户被洪水损毁房屋灾后重建，尽快恢复受灾群众的正常生产生活。积极应对人口老龄化，加快完善社会养老服务体系，动员社会力量兴办养老事业，全年新建日间照料服务中心451个、农村居家养老服务站226个，新增养老床位5000张，努力争创“全国养老服务业综合改革试点城市”。

推进医药卫生体制改革。统筹抓好医疗保障、医疗服务、公共卫生、药品供应、监管体制综合改革，积极探索建立合理分级诊疗模式，有效缓解百姓看病难、看病贵问题。力求提高医疗保障水平，启动实施城镇居民大病保险制度，二次报销比例不低于50%，统筹区域内新农合政策性报销补偿比例不低于80%。力求提高社区医疗服务能力，建立社区医生和居民契约服务关系，完善医疗机构与养老机构转诊合作机制，有序推进村卫生室和非政府办社区服务中心实施国家基本药物制度，实行零差价销售。力求完善公共卫生服务，免费为城乡居民提供11类43项基本公共卫生服务和8项重大公共卫生服务，努力让群众看病更方便、更满意。

促进文化大发展大繁荣。进一步完善文化管理体制，推动政府部门由办文化向管文化转变。深入实施文化惠民工程，继续抓好哈尔滨大剧院、音乐厅等重大公共文化基础设施建设，统筹推进市、区、街道（社区）和县、乡（镇）、村两个三级公共文化设施建设，支持南岗创建“全国公共文化服务体系建设示范区”。进一步提升文化发展层次和水平，广泛开展高雅艺术普及活动，着力打造百年交响乐团，高质量办好第32届“哈尔滨之夏”音乐会，不断满足广大人民群众日益增长的精神文化需要。切实加强历史文化名城保护，继续做好“731”和“金上京”遗址世界文化遗产申报工作，努力让历史文化资源得到更有效的保护和利用。大力发展文化产业，加快推进中国工美集团文化大世界、平房动漫基地等大项目建设，进一步提高文化产业规模化、集约化、专业化水平。发展体育事业，突出抓好“百万青少年上冰雪”活动，把冰雪项目纳入中招体育考试成绩；进一步扩大健身路径覆盖面，全力打造城区“10分钟健身圈”。加强精神文明建设，把工作着力点放在提升市民文明素质和创建全国文明城市上，全面落实全国文明城市和未成年人思想道德建设工作测评体系要求，实施工程式推进、常态化管理、品牌化运作、全社会参与，以更大的力度和显著的成效实现创城目标。

提高社会治理能力。深入实施“强基工程”，加大系统治理、依法治理、综合治理和源头治理力度，进一步完善以打击犯罪和确保交通、消防、生产、食品药品安全为重点的公共安全体系，持续打造全国最具安全感省会城市。提升社会服务功能，新建千米以上城市社区和农村示范社区20处。加强农村社会管理，健全农村留守儿童、留守妇女、留守老年人关爱服务体系。完善重大决策社会稳定风险评估、矛盾纠纷排查和“大调解”工作机制，及时预防和化解社会矛盾，确保人民安居乐业、社会安定有序。

继续做好广播电视、新闻出版、民族宗教、妇女儿童、残疾人、外事侨务、国防建设等工作，努力把哈尔滨各项事业不断推向前进！

三、加强政府自身建设

顺应改革发展新要求和人民群众新期待，全面贯彻落实党的十八届三中全会精神，扎实推进群众路线教育实践活动，着力打造法治政府、廉洁政府和服务型政府。

（一）解放思想，锐意改革。坚定改革信念。始终保持一种昂扬向上、敢为人先的精神状态，一种自我变革、舍利为公的思想境界，一种干就干好、一抓到底的工作韧劲，面对机遇锲而不舍，面对困难勇往直前，着力在创新体制机制、打造产业集群、统筹城乡发展、扩大沿边开放等重点工作上，先行先试、率先突破。加快创新推动。坚持问题导向，鼓励创新，宽容失败，以更大的勇气拼搏进取，以更大的智慧开拓创新，在破解项目建设要素瓶颈、构建新型农业经营体系、破除城市管理运行弊端等方面大胆地试、勇敢地闯，最大限度释放改革红利。聚集发展合力。认真接受人大依法监督和政协民主监督，充分发挥工会、共青团、妇联等人民团体桥梁纽带作用，在深化改革、扩大开放、完善社会治理、加强生态保护等重大事项上，增进共识、汇集力量，努力开创同心同德促改革、齐心协力抓发展的生动局面。

（二）勤政为民，真抓实干。立行立改转作风。始终把人民利益放在首位，严格落实中央和省、市委改进作风各项规定，着力解决人民群众反映强烈的突出问题，着力整治懒庸散奢等不良风气，不断把党的群众路线教育实践活动引向深入。大兴调查研究之风，深入基层、深入群众，问政于民、问需于民、问计于民，不断从广大人民群众实践中汲取智慧和力量。坚持艰苦奋斗、勤俭节约，严格财政预算管理和绩效管理，严控“三公”经费支出和楼堂馆所建设，把更多的财力用在改善民生、促进发展上。雷厉风行抓落实。坚持实干富民、实干兴邦，充分发扬“钉钉子”精神，一颗接着一颗钉、一锤接着一锤敲，凡是决策了的事项，凡是改革发展稳定重点任务，凡是百姓关注的重点难点问题，我们都要以踏石留印、抓铁有痕的作风，强化责任、一抓到底，确保抓出成效，确保说了算、定了干，言必信、行必果，积小胜为大胜，让百姓得到实实在在的好处，让全社会感受到市场环境、创业条件、干部作风在一天天好转。强化素质提能力。着力在公务员队伍中营造崇尚学习、勤思善为的良好风气，不断提升应对复杂局面、统筹驾驭全局的能力，抢抓政策机遇、推动更好更快发展的能力，研究问题、解决问题、破除瓶颈制约的能力，开拓进取、创新有为、推动工作落实的能力，切实解决责任心不强、心浮气躁、本领恐慌等突出问题，以能力提升、作风转变，促进经济社会加快发展。

（三）依法行政，创优环境。牢固树立法治理念。运用法治思维和法治方式思考处理问题，按照法定权限和程序履行职责，坚持依法科学民主决策，严格规范公正文明执法，让法治精神始终贯穿于政府工作全过程。深入开展“六五”普法，切实提高社会治理法制化水平。着力创优政务环境。完善政务公开制度，推进决策公开、服务公开、结果公开，促使公职人员恪守职业道德、自觉履职尽责，提供更周到、更便捷的服务。充分发挥便民服务监督中心、市长公开电话等平台作用，积极回应社会关切和百姓诉求，努力让企业和群众的合法权益得到更有效的保障。不断强化行政问责。继续开展“政风建设年”活动，严格执行行政问责和整治涉企“四乱”行为规定，重点规范基层科所队、专管员和一线执法人员行政行为，严厉查处破坏经济发展环境的人和事，持续唱响改善环境“好声音”，凝聚干事创业“正能量”。

（四）廉洁从政，干净干事。加强廉政教育。牢固树立正确的权力观、利益观和政绩观，筑牢思想防线，守住廉政底线，切实做到立身不忘做人之本、为政不移公仆之心、用权不谋一己之私。完善廉政制度。坚持用制度管权管事管人，健全反腐倡廉法规制度体系，加强廉政风险防控机制建设，落实“一岗双责”、“一案双查”，推行政府部门权力清单制度，努力构筑规范权力运行的制度屏障。强化廉政监督。重点加强对行政审批、项目招投标、土地出让、政府采购、国有资产产权交易等重点领域、关键环节的行政监察和审计监督，严肃查处各类违法违纪行为，坚决纠正损害群众利益的不正之风，确保让人民监督权力，让权力在阳光下运行。

各位代表，时代召唤我们前行，人民期待我们再创辉煌。让我们紧密地团结在以习近平同志为总书记的党中央周围，在省委、省政府和市委的正确领导下，紧紧依靠全市人民，励精图治、埋头苦干、奋发进取，为实现哈尔滨科学发展新跨越、谱写幸福美好生活新篇章而奋斗！
